# Real World Evidence: methodological issues and opportunities from the European Health Data Space

**DOI:** 10.1186/s12874-023-02014-3

**Published:** 2023-08-14

**Authors:** Stelios Kympouropoulos

**Affiliations:** 1 European Parliament, Altiero Spinelli, 09E240, 60 rue Wiertzstraat, Brussels, B-1047 Belgium; 2 Psychiatrist - Second Department of Psychiatry, University of Athens, ’ATTIKON’ University Hospital, Chaidari, Greece

**Keywords:** Real world data, Real world evidence, Health system, European Union

## Abstract

The current Evidence-Based-Medicine (EBM) approach is generally based on data coming from Randomized Clinical Trials or and, epidemiological observational studies. However, the past few years, with the explosion of available data derived from e-technology, a novel aspect regarding EBM arose, the Real-World-Data (RWD). RWD refers to data collected outside traditional studies, such as e-health records, claims data, patient-generated information, registries, etc. This type of information provides invaluable insights into the effectiveness, safety, and value of medical treatments and interventions when applied in real world settings. European Health Data Space (EHDS) is an initiative launched by the European Commission to create a secure and protected platform for exchanging health data across borders within European Union. The powerful combination of RWD within the EHDS serves as a valuable resource, supporting research initiatives. By analyzing diverse RWD sources, researchers generate Real-World Evidence (RWE) broadening medical knowledge. In this comment paper, methodological issues and opportunities of the application of EHDS in member states are discussed. Undoubtedly, EHDS creates a health-specific ecosystem empowering individuals through increased digital access and control their health data, providing a consistent, truthful and proficient set-up for the use of health data for research, innovation, policy-making and regulatory activities.

During the usual “*State of Union*” speech in September 2020, the President of European Commission, Ursula Von der Leyen, expressed the idea of the “European Health Union” after the first wave of Covid-19-related lockdowns and the unprecedented health situation faced. The health crisis due to the recent pandemic gave the opportunity for the European Union (EU) to better understand that the future needs to be united in the health sector, if Member-States want to have resilience. Therefore, the European Commission launched a variety of tools in order to fulfil this need. Among them, the European Health Data Space (EHDS) is a health-specific ecosystem proposal launched by the European Commission to create a secure and protected platform for exchanging health data across borders in EU [1]. In a detailed analysis, which allows for the identification of the key-barriers at EU as well as at national level, European Commission highlighted the causes and consequences for stakeholders of this massive exchange of data, and the potential evolution without EU intervention, and enabled a preliminary list of targeted areas for EU interventions [2].

We are living in a period where the availability of data has been updating with an extremely high speed; using only the inputs coming from Randomized Clinical Trials (RCT) or epidemiological, observational studies, is quite outdated [3]. Real-world data (RWD) hold a potential for generating robust evidence for designing and conducting confirmatory trials and answering questions that may not be addressed otherwise [4]. For example, RWD can ensure the trial’s relevance and representation of real-world clinical scenarios such as patient population or treatment patterns, aids in patient recruitment, safety monitoring, identifying adverse events promptly and among other things can enhance the generalizability and relevance of results in real-world healthcare settings.

Electronic health records, claims and billing data, patient registries, wearable device data, social media data, and data from mobile health applications can consist of sources of this new kind of collection. The result is that these different sources provide information on patients’ demographics, medical histories, treatment results, adverse events, medication adherence, lifestyle choices, and patient-reported outcomes. Through RWD, scientists may promote research and innovation in healthcare, while improving patients’ outcomes, too [5]. Real-World Evidence (RWE) refers to the evidence derived using these data and involves the analysis of RWD to generate insights and evidence about healthcare interventions and outcomes in real-world settings. This type of information provides invaluable insights into the effectiveness, safety, and value of medical treatments and interventions when applied in real world settings and can be a valuable collecting method to evaluate the effectiveness of a particular medication or treatment, assess the safety profile of interventions, compare different treatment strategies, or identify patient subgroups that may benefit most from specific therapies.

Real-World Evidence is utilized in several contexts to guide practice, influence policy, and propel progress. Some of the most important applications are as follows [6]:

i. For the purposes of pharmacovigilance and safety surveillance following the release of a drug, medical device, or intervention.
ii. Drug, device, and diagnostic test evaluations all benefit greatly as part of health technology assessment.
iii. Patient subgroups who may respond differently to treatments or interventions are identified through RWE, which contributes to the development of personalized medicine approaches.
iv. Better design and execute studies that answer unmet clinical needs, which in turn facilitates the recruitment of clinical cohorts likely to respond favorably to novel treatments.
v. The development of healthcare policies, clinical guidelines, and treatment pathways.

By harnessing the power of RWD, the EHDS facilitates the generation of RWE, which, in turn, becomes a crucial driver in supporting research, fostering innovation, and informing healthcare decision-making within the dynamic European healthcare ecosystem. Through the powerful combination of RWD and RWE, without diminishing the importance of RCTs, the EHDS becomes an instrumental resource in supporting research initiatives. Comparative effectiveness research flourishes as RWE allows researchers to evaluate the relative benefits and risks of various treatments and interventions in real-world settings. Moreover, researchers can analyze this wealth of information to generate evidence that reflects the complexities of routine clinical practice, ultimately complementing traditional clinical trial data and broadening the scope of medical knowledge. In addition, to supporting research and innovation, the EHDS can also aid in improving healthcare delivery and patients’ outcomes. This includes the benefits which can occur in rare disease research and patient care in Europe. In particular, the EHDS can help to facilitate the creation of rare disease registries, to improve rare disease diagnosis and treatment and to enhance patient engagement and participation in research. As we clearly understand, RWE can be utilized on the same direction through clinical decision-making, personalized treatment plans, as well as opportunities for quality improvement and cost savings within healthcare systems.

The EHDS was created as a response to the lack of effective data collection at EU level, in comparison with other non-European health environments. But along the way there have been several methodological challenges encountered; these include: Data Quality and standarisation; the EHDS will compile data from various sources, such as electronic health records, clinical registries and research databases, genomic databases and other real-world sources. Ensuring the quality of this information is crucial in order to guarantee research findings are valid and trustworthy. Additionally, defined data models are required to support data interoperability, making it easier for researchers and healthcare providers to exchange and use data across borders. Data Privacy and Security: the EHDS strives to create a secure environment for the exchange and sharing of health data across borders in the EU. However, safeguarding its privacy and security will be an immense task; there is potential for unauthorized individuals to access personal health records, leading to breaches in privacy as well as potential harm for individuals. Interoperability: the EHDS strives to facilitate interoperability of health data between various systems and sources. To make this goal attainable, common data standard must be created as well as technical infrastructure that facilitates data sharing and analysis. Unfortunately, this complex and resource-intensive process could take several years before completion. Methodological Heterogeneity: the EHDS will collect data from multiple sources, creating methodological heterogeneity. This makes it difficult to compare and combine findings across different sources and draw meaningful conclusions from research findings. As stated by Liu and Panagiotakos [4] analysis of RWD should be based on statistical criteria and inferential approaches that are adequate to incorporating the variety and complexity of RWD, as well as validating statistical hypotheses, and generating regulatory-grade RWE. Ethical Considerations: the EHDS will involve the use of personal health data, raising important ethical concerns. For instance, researchers must guarantee that data collection, management and analysis is done transparently and ethically while always safeguarding individuals’ privacy and confidentiality. Data sharing and collaboration are crucial for advancing research, but it is just as important to protect people’s privacy and autonomy over their own health data. Anonymization techniques play a critical role in addressing privacy concerns. However, as datasets grow in both size and variety ensuring the effectiveness of anonymization methods and mitigating re identification risks becomes challenging. Successfully tackling these challenges will require investments in data infrastructure, governance, privacy and security protections well as collaboration and coordination among various stakeholders and countries. Therefore, a pivotal question arises: *Are the Member States adequately prepared to confront these challenges?*

The readiness of Member States for the EHDS relies heavily on factors, including their existing health data infrastructure, policies and regulations related to data sharing and technical capabilities. Member-States that have already implemented health record systems and established policies or frameworks for sharing health data may be better positioned to participate in the EHDS. Conversely those lacking infrastructure, policies or technical capabilities may require support and investment to become part of this initiative. To address these disparities effectively the European Commission has allocated funding. Provided assistance to Member States in their preparation for the EHDS. For example, through its Horizon 2020 program it has financed research and innovation projects aimed at facilitating health data sharing while also developing infrastructure and standardized data formats. The Commission has also established a Governance Framework, for the EHDS to ensure that every Member State can participate regardless of their level of preparedness. This framework provides guidance and assistance to Member States on aspects such as data protection, cybersecurity, interoperability and ethical considerations among others.

In conclusion, the EHDS gives a substantial opportunity for RWE research in the health sciences throughout Europe, and not only. By facilitating the exchange and sharing of health data across EU countries the EHDS can support researchers in gaining insights and evidence to drive the development of treatments and technologies as well as enhance patient outcomes and overall population health. The voluminosity and complexity of RWD within the EHDS call for the development of more appropriate, sophisticated, and innovative data processing and methodological techniques. This is crucial for maintaining scientific rigor in research findings and ensuring attention to data ethics, ultimately harnessing the full power of RWD with EHDS. Additionally, protecting patients’ privacy and anonymity is a concern when utilizing health data for research purposes. The EHDS has implemented governance and data protection measures to address these issues ensuring that health data is collected, managed, and analyzed in a morally responsible manner. The potential benefits offered by the EHDS for health sciences research are substantial in areas like rare diseases research, personalized treatment approaches, timely access to therapies and patient centered care. However, it’s important to acknowledge that utilizing health data for research also poses challenges that need to be overcome. In order to surmount these challenges and unlock the capabilities of the EHDS in boosting health outcomes and advancing knowledge across Europe, it is crucial for various stakeholders, within the health sciences industry to join forces and work together.

## Data Availability

Not applicable.
